# Mental health impact on healthcare workers due to the COVID-19 pandemic: a U.S. cross-sectional survey study

**DOI:** 10.1186/s41687-022-00467-6

**Published:** 2022-06-13

**Authors:** Joshua Biber, Bethany Ranes, Shanieek Lawrence, Vishal Malpani, Trong Tony Trinh, Andrew Cyders, Steven English, Charles L. Staub, Kristen L. McCausland, Mark Kosinski, Nishtha Baranwal, Daniel Berg, Rodica Pop

**Affiliations:** 1 QualityMetric Incorporated, LLC, 1301 Atwood Avenue, Suite 216E, Johnston, RI 02919 USA; 2 OptumLabs, UnitedHealth Group, 5995 Opus Parkway, 5th Floor, Minnetonka, MN 55343 USA; 3 Optum Center for Research and Innovation (OCRI), UnitedHealth Group, 11000 Optum Circle, Eden Prairie, MN 55344 USA; 4 WellMed Medical Group, Optum Center for Research and Innovation (OCRI), UnitedHealth Group, 8637 Fredericksburg Road, San Antonio, TX 78240 USA; 5 The Polyclinic, Optum Center for Research and Innovation (OCRI), UnitedHealth Group, 904 7th Avenue, 2nd Floor, Seattle, WA 98104 USA; 6 The Everett Clinic, Optum Center for Research and Innovation (OCRI), UnitedHealth Group, 3901 Hoyt Avenue, Everett, WA 98201 USA

**Keywords:** COVID-19, Mental health, Healthcare workers

## Abstract

**Background:**

The COVID-19 pandemic has impacted the mental health and well-being of health care workers (HCWs). This study examined mental health outcomes and COVID-related stress impacts among a diverse sample of ambulatory HCWs, including clinicians and support staff, as well as the associations between mental health outcomes and work impairments in this population. Detailing these results can help in designing interventions to alleviate this burden.

**Methods:**

“The Health Care Worker Stress Survey” was administered to ambulatory care providers and support staff at three multispecialty care delivery organizations as part of an online, cross-sectional study conducted between June 8, 2020, and July 13, 2020.

**Results:**

The greatest stress impact reported by HCWs was the uncertainty regarding when the COVID-19 outbreak would be under control, while the least reported concern was about self-dying from COVID-19. Differences in COVID-19 stress impacts were observed by age, gender, and occupational risk factors. Approximately 50% of participants reported more than a minimal level of anxiety, including 22.5% who indicated moderate to severe levels of anxiety. Higher levels of anxiety were observed with younger ages and female gender, while occupational roles with increased exposure risk did not report higher levels of anxiety. Roughly two-thirds of the sample reported less than good sleep quality and one-third to one-half of the sample reported other sleep related problems that differed by age and gender. Role limitations due to emotional health correlated with COVID-19 related stress, anxiety and sleep problems.

**Conclusions:**

Using established, validated measures, we quantified mental health outcomes within a diverse sample of ambulatory care HCWs during the pandemic. Younger and female HCWs reported greater anxiety burden; HCWs with higher occupational risk of COVID exposure did not report higher levels of anxiety. Notable proportions of HCWs reported sleep and work impairments. Due to the cross-sectional nature of the study, it is difficult to attribute these patterns to the pandemic. These results underscore the depth and extent of mental health outcomes in HCWs in ambulatory settings and raise important questions on new interventions to relieve that burden. Further research is needed to study specific interventions to support the mental health and wellbeing of HCWs.

## Background

The COVID-19 pandemic has led to a global health crisis. Healthcare workers (HCWs) are on the front lines of this crisis, diagnosing and treating symptomatic patients and asymptomatic carriers of COVID-19. As frontline HCWs labor to treat patients, they also grapple with their own heightened exposure to COVID-19 infection and the resulting mental health stress. At the time of this study, there were only a handful of studies done on this topic, mainly coming from China. These studies identified significantly increased levels of depression, anxiety, and insomnia among HCWs [1]. Further research assessing the mental health impacts of COVID-19 on HCWs is imperative and can inform occupational health and workplace intervention programs focused on crisis management and improvement of the overall wellness of the health workforce.

COVID-19-induced distress among physicians, nurses, assistants and other HCWs can potentially disrupt the healthcare system. Evidence from previous epidemics shows that outbreaks can negatively impact the short and long-term mental health of HCWs significantly [2]. A study conducted two years after the SARS CoV-1 outbreak of 2003 revealed a 5% emergence of psychiatric disorders in HCWs [3]. Anxiety and feelings of social rejection due to potential exposure to the disease were also reported [4]. The Ebola Virus Disease outbreak in Sierra Leone (2014–2016) revealed an unprecedented infection rate in HCWs, who were 21–32 times more likely to be infected than the general adult population [5]. Interviews conducted with HCWs identified fear of contagion, concern for family health, interpersonal isolation, quarantine, and concerns surrounding trust and transparency of health organizations, as major stressors [6].

Perhaps the most demanding aspect of this current pandemic has been its pervasive uncertainty, particularly during the first year since its emergence. Uncertainty intolerance is strongly associated with the development of posttraumatic stress symptoms (PTSS) following an uncertain stressful event [7, 8] and has been identified among overworked HCWs prior to this pandemic [9]. In the early months of the pandemic there was uncertainty about the modes of transmission of this novel virus and methods to effectively reduce its spread. There was limited availability of testing, and neither vaccination nor disease specific pharmaceutical therapy was available at that time. Personal protective equipment shortages were widespread. With this background, working long hours in a clinical environment with increased risk of contracting COVID-19 infection contributed to elevated PTSS in various HCW populations around the world [10–12].

Elevated levels of burnout among medical professionals have been observed during this pandemic [13]. Burnout adversely impacts not only HCWs’ own physical and mental health causing increased disability and absenteeism, but also results in poorer patient outcomes, including medical errors and low patient satisfaction [14–16]. While certain mental health stressors are unique to the pandemic, many risk factors for mental distress and PTSS during COVID-19 are similar to the risks for HCW burnout identified prior to the pandemic. Salient factors include high workload, clinical uncertainty, and low levels of social support [9, 17]. Much of the current research in this area has focused on HCWs in hospital settings, but few studies have focused on HCWs in the outpatient environment.

National and international groups dedicated to protecting and promoting the safety, health, and wellbeing of workers emphasize the need for mental health initiatives targeting HCWs in the U.S. and abroad [18, 19]. To inform the development and continuous improvement of these mental health initiatives, valid and reliable methods for measuring HCW wellbeing are warranted. The use of validated instruments can help to provide better interpretation by classifying scores into meaningful groups using established thresholds or cut-offs and comparing existing results with benchmarks from previous studies. Furthermore, the use of baseline assessments can help to identify unmet needs, promote worker engagement, and provide an opportunity to measure change [20].

The objective of this study was to better understand the extent of mental health burden experienced by ambulatory HCWs during this pandemic using validated instruments to facilitate interpretation, to assess the association between mental health outcomes and the performance of work and other daily activities, and to examine the variation in mental health outcomes among subgroups of HCWs according to age, gender, and occupational risk.

## Methods

### Study design and sample

Data collection for an online, cross-sectional survey study, “The Health Care Worker Stress Survey”, was conducted between June 8, 2020, and July 13, 2020, at three separate multispecialty care delivery organizations: The Polyclinic in Seattle, WA, the Everett Clinic in Everett, WA and WellMed Medical Group in Texas and Florida. All employees and providers were eligible to participate. Physicians, Advanced Practice Clinicians (i.e., nurse practitioners and physician assistants), Nurses, Medical Assistants and Patient Service Representatives comprised the majority of the participants. There were no other inclusion or exclusion criteria; however, invites for the WellMed Medical Group were sent only to their physicians and advanced practice clinicians.

This study was reviewed and approved by the [UnitedHealth Group] IRB on 07 May 2020.

### Study procedures

All data were collected via anonymous surveys administered using a proprietary web portal. At the end of the survey, participants were given the option to contact the local behavioral health team (phone number provided) or the Employee Assistance Program if they needed guidance and/or support related to the stress caused by the COVID-19 pandemic.

### Measures

The study team worked collaboratively with staff members from the study sites to identify the most important concepts to include in the survey instrument. Next, the study team reviewed relevant literature and drew from previous survey experience to ensure the survey content could validly and reliably measure the constructs stated in the study objectives. In developing the final survey instrument, the study team considered whether the measures were practical and easily administered, provided readily interpretable results, and minimized respondent burden. The final survey included validated measures and novel survey items that assessed: anxiety, sleep patterns, COVID-19 related stress impacts, role limitations due to emotional problems, basic demographics and job information, and personal and occupational risk factors for COVID-19 exposure and/or outcomes.

#### Anxiety

The Generalized Anxiety Disorder-7 (GAD-7) is a 7-item, validated screener for generalized anxiety disorder based on the Diagnostic and Statistical Manual of Mental Disorders-IV (DSM-IV) criteria [21]. Each item asks respondents to characterize how frequently they experienced a specific symptom of anxiety during the past 2 weeks using 4 response options: “not at all”, “several days”, “more than half the days”, and “nearly daily”. Each item is scored on a scale of 0–3, with 3 representing a greater frequency of symptoms. Total sum scores range from 0 to 21 with higher scores representing greater levels of anxiety. For the current study, total sum scores were further classified into 4 levels of severity using established cut-offs (“minimal” (0–4); “mild” (5–9); “moderate” (10–14); “severe” (15–21) [21]. The GAD-7 has been validated in a variety of populations, including the general population and occupational health service settings [22].

#### Sleep patterns

Five survey items capturing different elements of sleep patterns were included in the current study. The first item asked study participants to report the average number of hours they slept each night during the past 4 weeks. This standard sleep duration item was identified from the sleep measures used in the Medical Outcomes Study [23]. The remaining items were chosen from the Patient-Reported Outcomes Measurement Information System (PROMIS) item banks for sleep-related impairment and sleep disturbance [24]. For each item, respondents rated aspects of their sleep during the past 7 days on a 5-point scale. To aid in interpretation, response categories for each item were collapsed during the analysis to create dichotomous variables representing the presence of each type of sleep pattern. Item-level analyses examining sleep patterns relied on these dichotomous variable specifications only; no overall scores were calculated. Participants who rated their sleep quality as “fair”, “poor” or “very poor” were classified as having poor sleep quality. Other dichotomous sleep patterns were specified by the following: *difficulty concentrating because of poor sleep* (defined as participants who reported they had a hard time concentrating because of poor sleep “somewhat”, “quite a bit”, or “very much” of the time); *difficulty falling asleep* (defined as participants who reported they laid in bed for hours waiting to fall asleep “sometimes”, “often”, or “always”); *difficulty staying asleep* (defined as participants who reported they woke up and had trouble falling back to sleep “sometimes”, “often”, or “always”).

#### COVID-related stress impacts

Thirteen survey items were created to evaluate COVID-19 related stress impacts. These items were adapted from a measure originally used to assess HCW stress during the 2014 Middle East Respiratory Syndrome Coronavirus (MERS-CoV) outbreak [25].To reduce respondent burden, the study team modified the original survey by reducing the number of items administered and revising the response options. For each item, participants were asked to report how much each stressor impacted their everyday life in the past 4 weeks using 5 response options (i.e., “not at all”, “a little”, “some”, “a lot”, and “extremely"). This deviated from the original survey where presence and severity of each stressor were evaluated using separate survey items. For the analysis in the current study, response options were collapsed to identify whether each participant was impacted “a lot” or “extremely” by each stressor during the past 4 weeks.

#### Role limitations due to emotional problems

Role limitations, or one’s ability to complete work or regular activities due to emotional health problems, was measured using 3 items from the SF-36v2® Health Survey (SF-36v2) Role-Emotional (RE) domain. The SF-36v2 is a generic measure of health-related quality of life that has been used widely in previous clinical- and population-based research [26].

For each item, study participants reported how frequently they had experienced a problem at work or during other regular daily activities due to emotional problems (such as feeling depressed or anxious) in the past 4 weeks. Each item had 5 response options ranging from “none of the time” to “all of the time”. Scores were calculated using the developer’s instructions and can range from 0 to 100 with higher scores representing better quality of life [26]. Scores were further standardized against a mean of 50 in the general population and a standard deviation (SD) of 10. The data from the U.S.-based normative sample were collected well before the COVID pandemic; therefore, the interpretation of the norm-based scores obtained in the current study are relative to a general population that was pandemic-naïve. This is in keeping with the standard interpretation of the SF-36v2 norm-based scores. To further aid in interpretation, a binary variable of RE Impairment was derived to classify individuals by whether or not they had a clinically meaningful deficit relative to the general population norm. A clinically meaningful deficit was defined as having a role emotional score that was ≥ 5 points (i.e., a half a SD) below the general population norm of 50.

#### Occupational risk factors for COVID-19 exposure

Several novel survey items were created to characterize participants’ personal and occupational risk factors for COVID-19 exposure or other outcomes. The occupational risk factors used in the present analyses included having direct patient contact and working in a respiratory clinical setting. HCWs who had direct patient contact were defined as those who reported working either in a hospital setting, an outpatient respiratory clinic or an outpatient non-respiratory clinic (all with direct patient contact). HCWs without direct patient contact may have worked onsite without interactions with patients, worked remotely, or provided telehealth care only. HCWs who reported working in a respiratory clinic were a subset of HCWs with direct patient contact.

### Statistical analyses

Descriptive analyses were conducted on the data collected from the online survey. Continuous variables were summarized using means, SDs, medians and range; categorical variables were summarized using frequencies and percentages. Departures from normality were assessed using graphical methods (histograms and quantile–quantile plots) and the Kolmogorov–Smirnov test on all applicable continuous variables.

The percentage of participants who reported “a lot” or “extreme” stress from each COVID-19 stressor were reported according to age, gender, and occupational risk groups (direct versus non-direct patient care and respiratory clinic versus non-respiratory clinic settings). Differences across groups were evaluated for statistical significance using chi-square analysis.

GAD-7 scores were not normally distributed; therefore, non-parametric tests were used to test for statistically significant differences in the distribution of scores across groups. The Kruskal–Wallis test was conducted to compare the distributions of GAD-7 scores across variables with more than 2 groupings (i.e., age groups); whereas the Wilcoxon two-sample tests were conducted to compare the distributions of GAD-7 scores for variables with only 2 groupings (i.e., gender and occupational risk factor groups). In addition, differences in the distribution of participants across GAD-7 severity groups by age, gender and occupation risk factor groups were evaluated for statistical significance using chi-square tests.

Average nightly sleep duration during the past 4 weeks was compared across age, gender and occupational risk factor groups and differences were assessed for statistical significance using a Kruskal Wallis test (age groups) and Wilcoxon two-sample tests (gender and occupational risk factor groups). In addition, the percentage of study participants reporting poor sleep quality or frequent sleep problems and disturbances was compared across age, gender and occupational risk groups. Differences across groups were assessed for statistical significance using the Chi-square test.

For the SF-36v2 RE scale, Student’s t-test was conducted to test the statistical significance of differences in mean scores between study participants and the normative score of 50 in the general US population. In addition, the percentage of study participants with significant role emotional impairment was compared across age, gender and occupational risk factor groups. Differences in the percentages across groups were assessed for statistical significance using the Chi-square test.

Data were analyzed using SAS 9.4.

## Results

The overall survey response rate for this study was 47.1%. Response rates ranged from 43.3% to 50.2% across the three multispecialty care delivery organizations. Participant demographics are displayed in Table 1. Of particular importance was that the majority (68%) of study participants had direct patient contact, increasing the chances of an individual being exposed to the virus and experiencing enhanced mental health impacts. The prevalence of direct patient contact varied across the different roles represented in the sample, with the greatest prevalence reported by physicians, advanced practice clinicians, and medical assistants/therapists (ranging from 90–93% within these groups), followed by nurses (81%) and patient service representatives (75%) (data not shown). Approximately two-thirds of lab service technicians (65%) reported direct patient contact compared to only a third of hospital administration employees (32%).

**Table 1:** Characteristics of study sample

Characteristic	N = 2606
*Age Group*	
18–34 years	656 (25.2%)
35–44 years	731 (28.1%)
45–59 years	899 (34.5%)
≥ 60 years	320 (12.3%)
*Gender*	
Male	536 (20.6%)
Female	1991 (76.4%)
Non-binary, third gender, or preferred not to answer	79 (3.0%)
*Role*	
Administration	265 (10.2%)
Advanced Practice Clinician (Nurse Practitioner / Physician’s Assistant)	308 (11.8%)
Lab Services (Lab Tech, Phlebotomist)	117 (4.5%)
Medical Assistant / Therapist	431 (16.5%)
Nurse (Registered Nurse, Licensed Vocational Nurse / Licensed Practical Nurse)	331 (12.7%)
Patient Service Representative / Technician	285 (10.9%)
Physician (Doctor of Medicine [MD] / Doctor of Osteopathic Medicine [DO])	486 (18.6%)
Other	383 (14.7%)
Personal risk factors for COVID-related complications (% yes)*	
Identifies as an individual at high risk	552 (21.2%)
Lives with children	1117 (42.9%)
Cares for someone that is high risk outside of home	313 (12.0%)
Changed living arrangements during COVID-19	219 (8.4%)
Occupational Risk Factors (% yes)*	
Works in respiratory clinical settings	364 (14.0%)
Has direct patient contact	1773 (68.0%)

^*^Percentages will not sum to 100 because these factors are pulled from distinct survey items, and participants were allowed to select more than one option

In examining a wide variety of potential COVID-related stressors, the greatest stress impact reported by HCWs was not knowing when the COVID-19 pandemic would be under control, with 42.2% reporting it impacted their daily life either “a lot” or “extremely” (Table 2). Participants also identified media coverage (35.0%) and needing to wear protective gear daily (34.8%) as stressors impacting their daily life. Approximately, a third of participants attributed a large stress impact on their daily lives to their concern of transmitting COVID-19 infection to a loved one or a loved one dying (34.1% and 32.1%, respectively). Conversely, less than half of participants were concerned about personally dying from COVID-19—52.3% attributed no impact on their daily lives due to this fear and only 9.1% reported “a lot” to an “extreme” impact. Significant differences were observed across age groups in the percentage reporting “a lot” or “extreme” impact on 10 of the 13 COVID-19 infection stressors. In general, the younger age groups showed a higher percentage reporting greater impact. Female HCWs experienced significantly greater impact across 5 of the 13 stressors as compared to males. Those HCWs involved with direct patient contact and working in a respiratory clinic were also more likely to report greater impact from COVID-19 stressors.

**Table 2:** Percentage of Healthcare Workers Reporting “A lot” or “Extreme” Stress Impacts of COVID-19 on Everyday Life

Stressor	Total sample	% Reporting “A lot” or “Extreme” stress impacts									
		Age groups				Gender		Occupational risk			
		18–34	35–44	45–59	≥ 60	Male	Female	Direct patient contact	Non-direct patient contact	Respiratory clinical setting	Non-respiratory clinical setting
Not knowing when the COVID-19 outbreak will be under control	42.2	50.3	44.8	38.7	29.4***	33.4	44.7***	43.3	37.3*	43.8	41.9
Media coverage of COVID-19	35.0	35.4	34.7	36.6	30.7	31.0	36.2	35.8	33.0	35.5	35.0
Need to wear protective gear on a daily basis	34.8	37.2	36.1	34.6	27.2*	28.0	36.3**	39.9	22.7***	45.2	33.1***
Concern about transmitting COVID-19 to family or friends	34.1	41.5	37.0	31.7	19.1***	29.5	35.2*	38.9	23.6***	46.1	32.2***
Concern about a loved one dying from COVID-19	32.1	39.8	31.2	30.9	21.3***	29.1	32.7	33.7	28.1*	37.4	31.2*
Lack of treatment for COVID-19	24.1	24.5	24.9	24.0	21.4	19.8	25.4*	24.3	23.3	25.3	23.9
Fear of financial difficulty due to job loss for self / significant other	23.8	30.5	27.4	20.5	11.3***	21.2	24.6	25.1	20.8	28.0	23.2*
Risk of contracting COVID-19 from a patient	22.1	25.9	23.1	21.4	14.4***	20.4	22.4	27.4	10.7***	31.7	20.6***
Seeing stressed or afraid colleagues	22.8	28.7	25.5	20.2	11.6***	19.6	23.3*	25.7	16.8***	31.8	21.3***
Conflict between duty and personal safety	18.4	23.1	19.6	16.8	10.9***	16.0	18.7	21.3	12.3***	24.2	17.5**
Worry that lapses in concentration could result in increased exposure to COVID-19 (self / others)	18.0	22.8	21.3	15.3	8.2***	18.1	18.0	20.1	13.8**	26.2	16.7***
Inadequate protective measures	12.9	17.3	13.4	11.0	7.8***	11.4	12.9*	15.2	7.8***	18.0	12.0**
Concern about self-dying from COVID-19	9.1	9.5	9.2	9.8	5.9	8.8	8.9	9.2	10.3	10.4	8.9

Statistical significance observed across/between groups based on a Chi-squared test

***p < 0.001

**p < 0.01

*p < 0.05

The mean GAD-7 score in this study sample was 5.96, which is just above the threshold (5 points) for minimal anxiety (Table 3). Approximately 50% of participants reported more than a minimal level of anxiety, including 22.6% of the sample who indicated moderate to severe levels of anxiety and 27.7% who indicated mild anxiety. Only 22.3% of the sample had a GAD-7 score indicative of no anxiety. Significant differences in the distributions of GAD-7 scores and GAD-7 severity levels were observed across age and gender groups. In general, the younger age groups experienced greater anxiety compared to the older age groups, and females experienced more anxiety than males. No difference in GAD-7 scores or severity levels were observed by occupational risk factors.

**Table 3:** Mean GAD-7 Score and Distribution of Anxiety Severity Levels among Healthcare Workers during the COVID Pandemic

	Total sample	Age groups				Gender		Occupational risk factors			
		18–34	35–44	45–59	≥ 60	Male	Female	Direct patient contact	Non-direct patient contact	Respiratory clinical setting	Non-respiratory clinical setting
*GAD-7 Total Score*											
Mean (SD)	5.96 (5.8)	7.44 (6.2)	6.32 (5.9)	5.32 (5.5)	3.92 (4.9)	5.06 (5.8)	6.15 (5.8)	6.03 (5.8)	5.72 (5.6)	6.21 (5.9)	5.92 (5.8)
Median	5.0	6.0	5.0	4.0	2.0***	3.0	5.0***	5.0	4.0	5.0	4.0
*Anxiety Severity (n, %)*											
None	581 (22.3)	108 (16.5)	144 (19.7)	224 (24.9)	105 (32.8)	168 (31.3)	399 (20.0)	379 (21.4)	100 (22.3)	81 (22.3)	500 (22.3)
Minimal	715 (27.4)	143 (21.8)	202 (27.6)	266 (29.6)	104 (32.5)	150 (28.0)	547 (27.5)	488 (27.5)	136 (30.3)	88 (24.2)	627 (28.0)
Mild	723 (27.7)	185 (28.2)	213 (29.1)	247 (27.5)	78 (24.4)	111 (20.7)	589 (29.6)	505 (28.5)	117 (26.1)	110 (30.2)	613 (27.3)
Moderate	297 (11.4)	115 (17.5)	80 (10.9)	82 (9.1)	20 (6.3)	58 (10.8)	229 (11.5)	198 (11.2)	54 (12.0)	38 (10.4)	259 (11.6)
Severe	290 (11.1)	105 (16.0)	92 (12.6)	80 (8.9)	13 (4.1)	49 (9.1)	227 (11.4)	203 (11.5)	42 (9.4)	47 (12.9)	243 (10.8)
Chi-Square, p-value	–	112.0, *p* < 0.001				38.3,* p* < 0.001		3.5, *p* = 0.475		4.1, *p* = 0.389	

The range for the GAD-7 scores was 0 – 21 overall and within each subgroup. Non-parametric tests were used to test for significant differences in the GAD-7 score distributions between subgroups. Wilcoxon two-sample tests were used to test for differences across categorical variables with two levels (i.e., gender and occupational risk factor groups). Kruskal–Wallis tests were used to test for differences across categorical variables with more than two levels (age groups). Chi-square tests were conducted to test for significant differences in the distribution of participants across GAD-7 severity groups by age, gender, and occupational risk factor groups

***p < 0.001

**p < 0.01

*p < 0.05

On average, HCWs reported 6.4 h a night of sleep during the past 4 weeks (Table 4). Average sleep duration differed significantly by age and type of patient care (direct vs. indirect); despite these statistical differences, all group means were within 20 min of each other; though medians differed by up to an hour. When assessing the quality of sleep, 67.1% of participants reported they had “fair”, “poor” or “very poor” sleep. Roughly a third of participants (30.6%) reported having difficulty concentrating because of poor sleep. Other sleep disturbances included difficulty falling asleep (36.0%) and difficulty staying asleep/disruptive sleep (50.4%). Differences in sleep quality and sleep disturbances differed by age and gender, but not by occupational risk factors. The younger age groups and females reported poorer sleep quality and greater frequency of sleep disturbances.

**Table 4:** Sleep Quality and Disturbances among Healthcare Workers during the COVID Pandemic

Sleep characteristic	Total sample	Age groups				Gender		Occupational risk factors			
		18–34	35–44	45–59	≥ 60	Male	Female	Direct patient contact	Non-direct patient contact	Respiratory clinical setting	Non-respiratory clinical setting
*Average nightly sleep duration during the past 4 weeks*											
Mean (SD)	6.4 (1.2)	6.3 (1.2)	6.4 (1.2)	6.4 (1.6)	6.6 (1.1)	6.5 (1.1)	6.4 (1.2)	6.4 (1.2)	6.5 (1.3)	6.3 (1.2)	6.4 (1.2)
Median (Range)	6.0 (2.0–14.0)	6.0 (3.0–12.0)	6.0 (2.0–10.0)	6.0 (3.0–14.0)	7.0** (3.0–9.0)	7.0 (2.0–9.0)	6.0* (2.0–14.0)	6.0 (2.0–14.0)	7.0* (3.0–12.0)	6.0 (3.0–9.0)	6.0 (2.0–14.0)
Sleep Patterns (% with pattern)											
Poor sleep quality	67.1	74.9	67.4	65.7	54.1***	57.1	69.7***	67.6	65.5	70.9	66.5
Difficulty concentrating because of poor sleep	30.6	42.7	32.0	26.4	14.7***	21.1	33.2***	31.1	30.3	32.1	30.4
Difficulty falling asleep	36.0	46.5	35.2	33.3	23.8***	23.7	39.1***	35.0	35.2	39.8	35.3
Difficulty staying asleep	50.4	51.2	49.4	52.8	43.8*	39.7	53.3***	49.9	50.3	53.0	49.9

Survey items assessing sleep characteristics were obtained from the PROMIS Sleep-Related Impairment and Sleep Disturbance item banks (Buysse et al., 2010). Non-parametric tests were used to test whether the distributions for average sleep time significantly differed between subgroups. Two outliers (i.e., values of 24) were removed from the analysis. Wilcoxon two-sample tests were used to test for differences in sleep time across categorical variables with two levels (i.e., gender and occupational risk factor groups). Kruskal–Wallis tests were used to test for differences across categorical variables with more than two levels (age groups). Chi-square tests were conducted to test for significant differences in the distribution of derived binary variables by age, gender, and occupational risk factor groups

***p < 0.001

**p < 0.01

*p < 0.05

On average, study participants scored significantly lower on the SF-36v2 RE scale compared to the general U.S. population norm (mean difference of 2.5, t = 9.6, *p* < 0.0001) (data not shown). Although statistically significant, this difference did not exceed the established threshold for a clinically meaningful group difference for the RE scale [26]. When considering the threshold for a clinically meaningful difference, nearly a third of study participants (29%) scored a half a standard deviation below the general population norm, indicative of a significant impairment in performing usual daily activities due to emotional health problems (Table 5). This proportion is comparable to what is typically seen in the general population given the norm-based scoring approach [26]. A greater percentage of HCWs in the younger age group experienced more role impairment due to emotional health than the older age groups. Similarly, a greater percentage of female HCWs experienced more role impairment than males. The differences in role impairment were not significant between occupational risk factor groups.

**Table 5:** Role Limitations due to Emotional Health (SF-36v2 RE) among Healthcare Workers during the COVID Pandemic

	Total Sample	Age groups				Gender		Occupational risk factors			
		18–34	35–44	45–59	≥ 60	Male	Female	Direct patient contact	Non-direct patient contact	Respiratory clinical setting	Non-respiratory clinical setting
% RE Impairment	29.0	40.9	30.1	24.6	14.4	20.7	30.7	29.7	28.1	33.2	28.3
		Χ^2^ = 87.0, *p* < 0.001				Χ^2^ = 20.8, *p* < = 0.001		Χ^2^ = 0.4, *p* = 0.505		Χ^2^ = 3.7, *p* = 0.053	

Chi-square tests were conducted to test for significant differences in the percentage of healthcare workers with RE impairment by age, gender, and occupational risk factor groups. RE Impairment was defined as having an RE score ≥ 5 points below the norm in the general U.S. population (Maruish et al., 2011)

Correlations between SF-36v2 RE scale scores and study participants’ responses to COVID-19 related stress, general anxiety and sleep problem items are presented in Table 6. All correlations were statistically significant (*p* < 0.001). Moderate (0.3 < r < 0.5) correlations were observed between the SF-36v2 RE scale and several COVID-19 related stress items, including not knowing when the outbreak would be under control (r = − 0.35), fear of financial difficulty due to job loss (r = − 0.31), conflict between duty and personal safety (r = − 0.34), seeing stressed or afraid colleagues (r = -0.37), worry about lapses in concentration that could result in exposure (r = − 0.36) and inadequate protective measures (r = − 0.31). Moderate correlations were also observed between the SF-36v2 RE scale and overall sleep quality rating (r = − 0.47) and other sleep pattern survey items: lying in bed for hours waiting to fall asleep (r = − 0.43) and woke up and had trouble falling back to sleep (r = − 0.42). Lastly, strong correlations were observed between the SF-36v2 RE scale and anxiety (as measured by the GAD-7 total score; r = − 0.62) and hard time concentrating because of poor sleep (r = − 0.59).

**Table 6:** Correlation between Role Limitations due to Emotional Problems (RE), COVID-19 Stressors, Anxiety and Sleep Problems

	Correlation with SF-36v2 RE scale *r*
*COVID-19 Stressors*	
Not knowing when the COVID-19 outbreak will be under control	**− 0.34**
Media coverage of COVID-19	− 0.27
Need to wear protective gear on a daily basis	− 0.26
Concern about transmitting COVID-19 to family or friends	− 0.27
Concern about a loved one dying from COVID-19	− 0.29
Lack of treatment for COVID-19	− 0.27
Fear of financial difficulty due to job loss for self / significant other	**− 0.31**
Risk of contracting COVID-19 from a patient	− 0.26
Seeing stressed or afraid colleagues	**− 0.37**
Conflict between duty and personal safety	**− 0.34**
Worry lapses in concentration could result in increased exposure	**− 0.36**
Inadequate protective measures	**− 0.31**
Concern about self-dying from COVID-19	− 0.25
*Anxiety (GAD-7)*	**− 0.62**
*Sleep Problems*	
My sleep quality was…	**− 0.47**
Hard time concentrating because of poor sleep	**− 0.59**
Laid in bed for hours waiting to fall asleep	**− 0.43**
Woke up and had trouble falling back to sleep	− **0.42**

All correlations statistically significant (*p* < 0.001). Bolded coefficients indicate meaningful associations

## Discussion

This study presents patterns of mental health outcomes across a diverse group of HCWs working in ambulatory healthcare settings located in 3 states in the United States during the COVID-19 pandemic. The timeframe of the survey coincided with a period when case numbers in all 3 states were rising along with a global shortage of personal protective equipment (PPE) [27, 28]. While most studies address the impact of COVID-19 on hospital healthcare workers, our study importantly includes outpatient providers, clinical staff, and non-clinical personnel.

Our study confirms a high level of anxiety among outpatient HCWs and provides information on the associations between anxiety and HCW characteristics, including gender, working role and age. Approximately half of the participants reported a detectable level of anxiety. These findings are aligned with most published studies reporting anxiety in HCWs during this period [29, 30].The prevalence of moderate and severe anxiety in the present study (22.5%) was markedly higher than the reported 5% among the general population prior to the pandemic [12]. Our study findings are similar to COVID-19 related levels of anxiety reported among HCWs in Asia and U.S. essential retail employees in Boston (24%) [11]; however, this study reveals a lower rate of anxiety than a European study which showed that 40% of frontline doctors had at least moderate anxiety as measured by the GAD-7 [15].

The prevalence and severity of anxiety in our sample differed by age and gender. About 33.5% of the participants between the ages of 18 and 34 years reported moderate to severe anxiety compared to only 10.4% of those 60 years or older. Similarly, 61.7% of the younger participants reported at least mild signs of anxiety compared to only 34.8% in the older group. While these findings are comparable to those from other COVID-19 related studies [14], they are contrary to what one may expect given higher fatality risks of COVID-19 infection among older adults. Previous studies have reported lower burnout in late career physicians, possibly due to reduced work hours, increased administrative activity, and better satisfaction with work life balance [31]. Reported reasons for increased anxiety among younger adults might include their use of social media and an increase in economic challenges facing younger people during this time [32], as well as the demands of childcare and schooling at home. The observed proportion of male and female participants reporting any signs of anxiety aligns with other current studies of HCWs, but with higher rates [33]; 52.5% of female participants and only 40.6% of male participants reported signs of anxiety, compared to results from a recent COVID-19 meta-analysis where the prevalence of anxiety was 29% for females and 20.9% for males. A higher incidence of anxiety experienced by female HCWs is reported in other studies and may be fueled by the additional burden of caring for family and children [33]. Gender differences in our study were less significant for moderate and severe anxiety (19.9% males vs 22.9% females) [29, 30].

We found no significant associations in our study between anxiety and occupational risk factors of COVID-19 exposures, such as having direct patient contact and/or working in a respiratory clinic, while there was some evidence of increased stressor prevalence in this subgroup. These findings contrast with other published studies, where HCWs working directly with or caring for COVID-19 patients reported higher levels of anxiety [29]. Our survey participants primarily treated outpatients screened to exclude patients with respiratory symptoms, except for those working in the respiratory clinic where patients with possible COVID-19 were treated. These factors may help account for the difference in anxiety among patient-facing HCWs in our study compared with others studying hospital-based HCWs.

Although anxiety levels did not differ by occupational risk factors, there were differences in the impacts of COVID-related stressors and patterns of sleep. The greatest stress impact among participants was due to the uncertainty of the pandemic, with 42.2% of the participants reporting “a lot” or “extreme” levels of stress associated with not knowing when the pandemic will be under control. Other high-level stressors included possibly transmitting COVID-19 to a loved one and/or seeing a loved one dying from COVID-19. This is similar to results from a previous study in response to the 2014 MERS-CoV outbreak in Saudi Arabia [25]. The top stressors reported in that study were seeing colleagues being intubated (96%), possibly transmitting the virus to family or friends (94%), and not knowing when the outbreak would be under control (91%). Carrying the infection home and possibly infecting a loved one and the fear of infecting a coworker (72.5%) were commonly reported as stressors and a source of anxiety in recent studies and meta-analyses [34, 35]. Interestingly in our study, HCWs were more concerned with transmitting the infection to others than in dying themselves from COVID-19. These results may suggest that a commitment to duty or other factors that contribute to the increased anxiety around the safety of others compared to one’s own safety. Stressor impact also varied by age, with the most significant differences noted between those 18–34 years old and those 60 years old and older, and for stressors related to the uncertainty of when the pandemic would end, concern about transmitting the virus to family or friends or loved ones dying from COVID-19, fear of financial difficulty, and seeing stressed/fearful colleagues.

In addition to providing information on anxiety, our study provides further data quantifying sleep disturbances in HCWs. Short sleep durations (6 or fewer hours of sleep per night) were more prevalent in the HCWs who participated in the present study compared to the general US population pre-COVID (50% vs. 32.9%) [36]. Poor sleep quality was common, reported by 67% of study participants. About a third also reported difficulty concentrating due to poor sleep. Sleep disturbances among HCWs during the pandemic are reported in several other studies and meta-analyses with variations by age and profession [34]. In a recent study in China during the pandemic, healthcare professionals reported rates of sleep disturbances as high as 66.1% [37]. Female participants in our study and those in the younger age groups experienced a higher incidence of sleep disturbances. Higher incidence of sleep disturbances related to COVID-19 in female HCWs were also reported in a study conducted in Bahrain [38] and a review of literature [33].

This study provided an important step towards quantifying the anxiety and sleep burden in this population during the pandemic. Given the cross-sectional nature of this study, we could not directly examine whether sleep, anxiety and stress worsened during the pandemic and whether these outcomes directly impacted HCW’s work performance. Despite this limitation, a notable proportion of the sample (30%) had SF-36v2 RE scale scores that were meaningfully lower than the general population norm – indicative of a significant impact of emotional health on the ability to perform usual daily activities, such as work. While this proportion is likely comparable to the prevalence of role-emotional impairments found in the general population prior to the pandemic, it underscores the critical need to protect and promote the wellbeing of HCWs and to understand how HCW wellbeing can impact downstream outcomes such as work performance and patients’ care.

## Limitations

Our study was cross-sectional and descriptive in nature, limiting our ability to make temporal or causal inferences related to the observed associations. Individuals with a history of pre-existing mental health conditions may be at a greater risk of relapse or worsening of symptoms due to the stress of the COVID-19 outbreak. Without accounting for participants’ history of anxiety or sleep impairments, we cannot accurately assess whether a change occurred and whether the current state is attributable to the pandemic. Despite this limitation, the use of validated measures and comparisons to other benchmarks helped to contextualize the results. The use of widely used measures of mental health outcomes, such as the GAD-7, the SF-36v2, and PROMIS items, show promise in identifying unmet needs in the workforce and was a strength to this study. Using a standardized measurement approach that is easily administered and interpretable can provide efficiencies and inform priority-setting for a range of stakeholders, including researchers, policymakers, and employers, who play critical roles in designing and/or funding mental health initiatives for HCWs.

## Conclusions

Our survey helps to clarify mental health outcomes in a large and broad spectrum of HCWs delivering outpatient care in the United States during the early months of the COVID-19 pandemic. Results demonstrated a high level of anxiety among outpatient HCWs and provided information on variations by gender, working role and age. This information can be used to inform employer groups and other stakeholders in the delivery of health care. Further research is needed to identify the root causes of the differences observed by gender and age, to better understand the complex relationships between these mental health impacts, and to use this information to design interventions to support HCWs during this pandemic and in other future periods of severe stress.

## Data Availability

The datasets analyzed in this study are not publicly available.

## Abbreviations

GAD-7: Generalized Anxiety Disorder 7 Item
HCW: Healthcare worker
PTSS: Posttraumatic stress symptoms
PROs: Patient-reported outcome measurement information system
RE: Role emotional
SF-36v2: Health survey

## References

[1] Lai J, Ma S, Wang Y, Cai Z, Hu J, Wei N (2020). Factors associated with mental health outcomes among health care workers exposed to coronavirus disease 2019. JAMA Netw Open.

[2] Maunder RG, Lancee WJ, Balderson KE, Bennett JP, Borgundvaag B, Evans S (2006). Long-term psychological and occupational effects of providing hospital healthcare during SARS outbreak. Emerg Infect Dis.

[3] Lancee WJ, Maunder RG, Goldbloom DS, Coauthors for the Impact of SARS Study (2008) Prevalence of psychiatric disorders among Toronto hospital workers one to two years after the SARS outbreak. Psychiatric Serv (Washington, D.C.) 59(1):91–95.10.1176/ps.2008.59.1.91. 10.1176/ps.2008.59.1.91 PMC2923654 18182545

[4] Brooks SK, Dunn R, Amlôt R, Rubin GJ, Greenberg N (2018). A systematic, thematic review of social and occupational factors associated with psychological outcomes in healthcare employees during an infectious disease outbreak. J Occup Environ Med.

[5] World Health Organization (2015) Health worker Ebola infections in Guinea, Liberia, and Sierra Leone—a preliminary report. Geneva, Switzerland

[6] Raven J, Wurie H, Witter S (2018). Health workers’ experiences of coping with the Ebola epidemic in Sierra Leone’s health system: a qualitative study. BMC Health Serv Res.

[7] Oglesby ME, Boffa JW, Short NA, Raines AM, Schmidt NB (2016). Intolerance of uncertainty as a predictor of post-traumatic stress symptoms following a traumatic event. J Anxiety Disord.

[8] Oglesby ME, Gibby BA, Mathes BM, Short NA, Schmidt NB (2017). Intolerance of uncertainty and post-traumatic stress symptoms: an investigation within a treatment seeking trauma-exposed sample. Compr Psychiatry.

[9] Kuhn G, Goldberg R, Compton S (2009). Tolerance for uncertainty, burnout, and satisfaction with the career of emergency medicine. Ann Emerg Med.

[10] Carmassi C, Foghi C, Dell'Oste V, Cordone A, Bertelloni CA, Bui E (2020). PTSD symptoms in healthcare workers facing the three coronavirus outbreaks: what can we expect after the COVID-19 pandemic. Psychiatry Res.

[11] Liu N, Zhang F, Wei C, Jia Y, Shang Z, Sun L (2020). Prevalence and predictors of PTSS during COVID-19 outbreak in China hardest-hit areas: Gender differences matter. Psychiatry Res.

[12] Shechter A, Diaz F, Moise N, Anstey DE, Ye S, Agarwal S (2020). Psychological distress, coping behaviors, and preferences for support among New York healthcare workers during the COVID-19 pandemic. Gen Hosp Psychiatry.

[13] Jalili M, Niroomand M, Hadavand F, Zeinali K, Fotouhi A (2020) Burnout among healthcare professionals during COVID-19 pandemic: a cross-sectional study. medRxiv, 2020.06.12.20129650. 10.1101/2020.06.12.20129650. 10.1007/s00420-021-01695-x PMC8052946 33864490

[14] Patel RS, Bachu R, Adikey A, Malik M, Shah M (2018). Factors related to physician burnout and its consequences: a review. Behav Sci (Basel, Switzerland).

[15] Salvagioni DAJ, Melanda FN, Mesas AE, González AD, Gabani FL, de Andrade SM (2017). Physical, psychological and occupational consequences of job burnout: a systematic review of prospective studies. PLoS ONE.

[16] Panagioti M, Geraghty K, Johnson J, Zhou A, Panagopoulou E, Chew-Graham C (2018). Association between physician burnout and patient safety, professionalism, and patient satisfaction: a systematic review and meta-analysis. JAMA Intern Med.

[17] Bria M, Baban A, Dumitrascu DL (2012). Systematic review of burnout risk factors among European healthcare professionals. Cognit Brain Behav Interdiscip J.

[18] U.S. Department of Health and Human Services (2021). HHS Health Workforce Strategic Plan. https://bhw.hrsa.gov/sites/default/files/bureau-health-workforce/about-us/hhs-health-workforce-strategic-plan-2021.pdf

[19] World Health Organization (2016). Working for health and growth: investing in the health workforce.: Report of the High-Level Commission on Health Employment and Economic Growth. Geneva, Switzerland.

[20] National Institute for Occupational Safety and Health (NIOSH) (2016). Fundamentals of total worker health approaches: essential elements for advancing worker safety, health, and well-being. Cincinnati, OH.

[21] Spitzer RL, Kroenke K, Williams JBW, Löwe B (2006). A brief measure for assessing generalized anxiety disorder: the GAD-7. Arch Intern Med.

[22] Plummer F, Manea L, Trepel D, McMillan D (2016). Screening for anxiety disorders with the GAD-7 and GAD-2: a systematic review and diagnostic metaanalysis. Gen Hosp Psychiatry.

[23] Hays RD, Stewart AL, Stewart AL, Ware JE (1992). Sleep measures. Measuring functioning and well-being: the Medical Outcomes Study approach.

[24] Buysse DJ, Yu L, Moul DE, Germain A, Stover A, Dodds NE (2010). Development and validation of patient-reported outcome measures for sleep disturbance and sleep-related impairments. Sleep.

[25] Khalid I, Khalid TJ, Qabajah MR, Barnard AG, Qushmaq IA (2016). Healthcare workers emotions, perceived stressors and coping strategies during a MERS-CoV outbreak. Clin Med Res.

[26] Maruish ME, Kosinski M, Bjorner JB, Gandek B, Turner-Bowker DM, Ware JE (2011) User’s manual for the SF36v2 Health Survey

[27] Burki T (2020). Global shortage of personal protective equipment. Lancet Infect Dis.

[28] Centers for Disease Control and PRevention. COVID Data Tracker: Trends in Number of COVID-19 Cases and Deaths in the US Reproted to CDC, by State/Territory.

[29] Malik A, Hafeez MM, Waquar S, Rana MA, Alam R (2020). Anxiety levels among healthcare professionals during COVID-19 pandemic: a multifactorial study. medRxiv. 10.1101/2020.10.14.20212167.

[30] Pappa S, Ntella V, Giannakas T, Giannakoulis VG, Papoutsi E, Katsaounou P (2020). Prevalence of depression, anxiety, and insomnia among healthcare workers during the COVID-19 pandemic: a systematic review and meta-analysis. Brain Behav Immun.

[31] Dyrbye LN, Varkey P, Boone SL, Satele DV, Sloan JA, Shanafelt TD (2013). Physician satisfaction and burnout at different career stages. Mayo Clin Proc.

[32] Salari N, Hosseinian-Far A, Jalali R, Vaisi-Raygani A, Rasoulpoor S, Mohammadi M (2020). Prevalence of stress, anxiety, depression among the general population during the COVID-19 pandemic: a systematic review and meta-analysis. Glob Health.

[33] Shaukat N, Ali DM, Razzak J (2020). Physical and mental health impacts of COVID-19 on healthcare workers: a scoping review. Int J Emerg Med.

[34] da Silva FCT, Neto MLR (2021). Psychiatric symptomatology associated with depression, anxiety, distress, and insomnia in health professionals working in patients affected by COVID-19: a systematic review with meta-analysis. Prog Neuropsychopharmacol Biol Psychiatry.

[35] Shanafelt T, Ripp J, Trockel M (2020). Understanding and addressing sources of anxiety among health care professionals during the COVID-19 pandemic. JAMA.

[36] Sheehan CM, Frochen SE, Walsemann KM, Ailshire JA (2019) Are U.S. adults reporting less sleep?: Findings from sleep duration trends in the National Health Interview Survey, 2004–2017. Sleep. 10.1093/sleep/zsy221 10.1093/sleep/zsy221 PMC6941709 30452725

[37] Wang W, Song W, Xia Z, He Y, Tang L, Hou J (2020). Sleep disturbance and psychological profiles of medical staff and non-medical staff during the early outbreak of COVID-19 in Hubei Province, China. Front Psych.

[38] Jahrami H, BaHammam AS, AlGahtani H, Ebrahim A, Faris M, AlEid K et al (2020) The examination of sleep quality for frontline healthcare workers during the outbreak of COVID-19. Sleep & Breathing = Schlaf & Atmung. 10.1007/s11325-020-02135-9. 10.1007/s11325-020-02135-9 PMC7319604 32592021

